# Influence of Operator, Tool, Dental Loupes, and Tooth Position on Enamel Loss and Composite Remnants After Removal of Composite Attachments for Orthodontic Clear Aligners: An Experimental Study Using 3D Profilometry

**DOI:** 10.3290/j.jad.b5876265

**Published:** 2024-12-10

**Authors:** Juliette Vandeloise, Adelin Albert, Raphael Herman, Maher Eldafrawy, Christelle Sanchez, Laurence Seidel, Annick Bruwier, Amélie Mainjot

**Affiliations:** a Orthodontist, Department of Orthodontics and Dentofacial Orthopedics, University of Liège (ULiège) and University of Liège Hospital (CHU), Liège, Belgium. Acquisition, analysis, and interpretation of data, and has drafted the manuscript.; b Professor Emeritus of Biostatistics, Biostatistics and Research Methods (B-STAT), University Hospital of Liège, University of Liège, Liège, Belgium. Conception and design of the work, analysis, and interpretation of data, and revised the manuscript.; c Laboratory Technician, Dental Biomaterials Research Unit (d-BRU), University of Liège (ULiège), Liège, Belgium. Acquisition and analysis of data and has revised the manuscript.; d Dentist, Dental Biomaterials Research Unit (d-BRU), University of Liège (ULiège), Liège, Belgium. Acquisition and analysis of data, and revised the manuscript.; e Senior Scientist, Dental Biomaterials Research Unit (d-BRU), University of Liège (ULiège), Liège, Belgium; Department of Fixed Prosthodontics, Institute of Dentistry, University of Liège (ULiège) and University of Liège Hospital (CHU), Liège, Belgium. Analysis and interpretation of data, and revised the manuscript.; f Biostatistician, Biostatistics and Research Methods (B-STAT), University Hospital of Liège, University of Liège, Liège, Belgium. Analysis and interpretation of data, and revised the manuscript.; g Professor of Orthodontics, Department of Orthodontics and Dentofacial Ortho-pedics, University of Liège (ULiège) and University of Liège Hospital (CHU), Liège, Belgium; Dental Biomaterials Research Unit (d-BRU), University of Liège (ULiège), Liège, Belgium. Conception of the work, acquisition of data and has revised the manuscript.; h Professor of Dental Biomaterials, Dental Biomaterials Research Unit (d-BRU), University of Liège (ULiège), Liège, Belgium; Department of Fixed Prosthodontics, Institute of Dentistry, University of Liège (ULiège) and University of Liège Hospital. Conception and design of the work, analysis, and interpretation of data, and has drafted the manuscript.; * Contributed equally to this work and share senior authorship.

**Keywords:** auxiliaries, Easycomp, magnification, orthodontics, Smoozies

## Abstract

**Purpose:**

To assess the influence of operator, tool, dental loupes, and tooth position on enamel loss and composite remnants after removal of composite attachments (CA) for orthodontic clear aligners. Procedure duration was also analyzed.

**Materials and Methods:**

Eight maxillary resin dental arches with four natural teeth were placed in the right posterior sector in dental simulators, and CA was realized. The dental arches were randomly distributed according to three experimental factors: operator (junior, senior), tool (tungsten carbide bur and silicone polisher, only silicone polishers), and use of dental loupes. Dental arches were scanned with 3D profilometry before and after CA removal to measure enamel surface height variation (ESHV), particularly enamel loss in the CA area. Digital microscopy was used to detect composite remnants.

**Results:**

The mean enamel loss was –22.7 ± 29.4 µm (range –132 to 0 µm). It was not significantly influenced by experimental factors or tooth position. Composite remnants were found in 34.4% of teeth, significantly more in senior than in junior operators (p = 0.038). They were more frequent with silicone polishers than with tungsten carbide burs (p = 0.0005) and were reduced using dental loupes (p = 0.0090). Junior operators worked faster than senior operators (p = 0.031), but the latter were quicker when using the dental loupes (p = 0.012).

**Conclusion:**

Aligner CA removal induces enamel damage or leaves composite remnants on its surface. The presence of composite remnants is influenced by the type of tool and can be reduced by using dental loupes, which also lowers working time.

Aligner treatments have become an important part of the orthodontists’ therapeutic arsenal in the past two decades. Dental movement possibilities have been greatly improved in recent years with the introduction of composite attachments (CA).^19^ This requires the enamel surface to be treated with a dental adhesive system and attachments to be molded on teeth using dental flowable composite. However, removing these attachments at the end of the orthodontic treatment remains a challenge due to the risk of damaging the enamel and of potential presence of composite remnants.

Enamel damage has been studied in the context of orthodontic brackets debonding. Brackets are bonded to the enamel surface using high-viscosity composite resin cement. The enamel damage after removal of the resin composite cement is believed to vary with the protocol used. The most recommended protocol is the use of a tungsten carbide bur, using silicone polishers to remove the last layer of composite resin cement.^1,3,5,7,12,17, 18,20,23,27,28,30,31^ However, some tools, like diamond burs or airborne-particle abrasion, were shown to be aggressive toward the enamel surface.^7,30^ In fact, all tools were reported to induce enamel damage (creation of grooves on the surface or enamel loss), but protocols using tungsten carbide bur proved to be less invasive.^1,5,11,17,20,23,27,28,31^ On the other hand, the persistence of composite resin cement on the tooth surface after debonding is also of concern. Indeed, those composite remnants are submitted to aging and can show shade alteration with time leading to esthetic impairment.^6^ According to Baumann et al, the use of dental loupes can markedly reduce the resin composite cement residues after orthodontic bracket removal.^3^

To date, the enamel damage caused by the removal of aligner CA has not been studied. Yet, the clinical situation and the composite materials used are different compared to orthodontics brackets. Indeed, in the context of aligners, a thicker layer of material, which is a flowable composite, will have to be removed. It can take more time, and the procedure can be complicated by the fact that, during the molding process, the use of flowable composite results in fine excesses that flow around the attachment and are difficult to visualize because they are tooth-colored. Consequently, enamel damage and the presence of composite remnants after the removal procedure of CA require the development of specific studies.

Different methods have been developed to study enamel surface modification after surface treatment. The use of scanning electron microscope (SEM) combined with the use of indexes, such as ESI (enamel surface index), SRI (surface roughness index), or EDI (enamel damage index), allows for a qualitative assessment of enamel surface modification and roughness.^2,3,12,18,28,30^ Most of the studies on orthodontic brackets debonding use this technique but do not allow for a quantitative evaluation of enamel loss. On the other hand, several *in vitro* studies analyzed surface roughness changes with methods such as contact profilometry,^1,7,8,10,25,27^ atomic force microscopy,^14,17^ or non-contact scanning white-light interferometry.^5^ Only one study reported enamel loss with tungsten carbide bur versus diamond disks using contact profilometry, which has only limited resolution.^27^

The objective of this experimental study was twofold: (1) to use 3D-profilometry to assess the influence of operator, tool, dental loupes, and tooth position on enamel loss after removal of CA for orthodontic clear aligners; (2) to study the influence of these factors on the presence of composite remnants and on procedure duration.

## Materials and Methods

### Experimental Design

The study was approved by the ethics committee of the (Blinded) on residual human material obtained after informed patient consent. The study was based on a factorial experimental design in which 8 presumably identical dental arches with 4 teeth (3 molars and 1 premolar) were randomly allocated to four operators (2 juniors and 2 seniors), each operator receiving 2 dental arches; 1 arch was treated by tungsten carbide bur and silicone polisher and the other one by only silicone polishers. Dental loupes were randomly assigned to one junior operator and one senior operator for each tool. Thus, all 4 teeth of the same dental arch were associated with the same operator (junior or senior), tool (tungsten carbide bur or silicone polishers only), and dental loupe (with or without).

### Preparation of Dental Arches

Extracted premolars and molars were used. The inclusion criteria were the absence of decay, composite fillings, and enamel lesions such as hypoplasia or hyper-mineralization. Teeth were stored in distilled water at 4°C to avoid any bacterial proliferation. Water was renewed every week. Teeth were then positioned in resin dental arches, in which 3 molars and 1 premolar were embedded in the right posterior sector of each maxillary arch.^22^

### Realization of Composite Attachments

**Fig 1:**
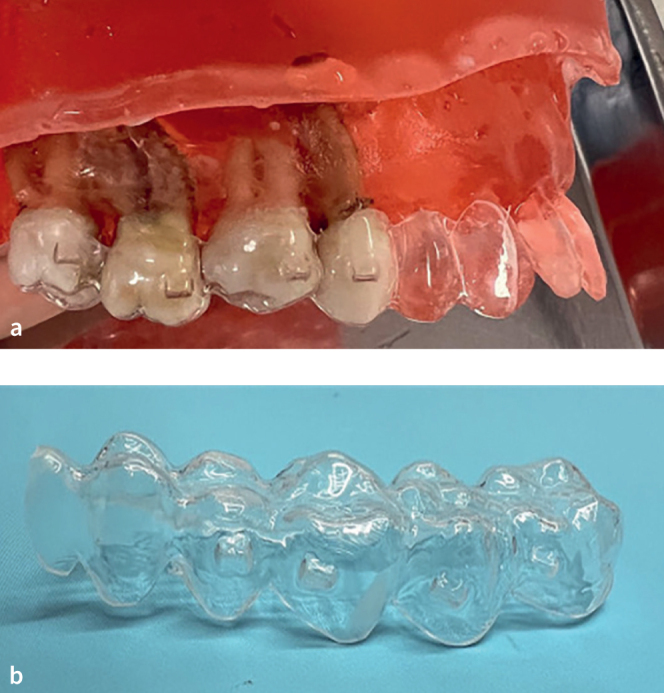
*(a)* Resin dental arch including 3 molars and 1 premolar. *(b)* Customized template.

Composite attachments were realized following the manufacturer recommendations by the same operator (JV). The arches were scanned using the Itero scanner (Itero 5, Align Technology). Customized templates were then CAD-CAM manufactured for each arch to create a CA on the buccal surface of each tooth (Fig 1). These templates were made in low-density polyethylene and were 0.75 mm thick. The CA was placed on the mesial cusp of the molars. For the premolars, the CA was positioned in the center of the vestibular surface. The teeth were cleaned with water and fluoride-free pumice (Zircate Prophy Paste, Dentsply Sirona, Milford, Germany) with a prophylactic brush (Hawe Prophy-Cup Latch-Type, KerrHawe SA, Bioggio, Switzerland), rinsed with water and dried with an air syringe. The CA area was then etched with 37% H_3_PO_4_ for 15 s, thoroughly rinsed with an oil-free air-water spray for 15 s, and air-dried until the enamel appeared chalky white. Bonding adhesive (Scotchbond Universal Adhesive, 3M Unitek, Germany) was applied with a micro brush for 20 s, air-dried for 5 s, and light-cured for 10 s. The CA were then molded in flowable composite (Filtek Supreme Ultra flowable, 3M Unitek), filling the customized template, placing it on the corresponding arch, and light-curing the composite for 12 s (6 s on the mesial side and 6 s on the distal side) (Ortholux, 3M Unitek, 430–480 nm light output).

### Removal of the Composite Attachments

To mimic a real clinical environment, the dental arches were inserted into dental simulators (Phantom Head PK2, Frasaco, Tettnang, Germany). The two junior operators were postgraduate students in orthodontics, and the two senior operators were specialists in orthodontics at the University Hospital Center. Each operator used two different tools, respectively the Smoozies kit (Komet, Paris, France) (S-kit) and the Easycomp kit (Easycomp RA, EY-1, Eve, Keltern, Germany) (E-kit), with or without dental loupes (2.5× magnification) according to the experimental design. For the S-kit, a first multiblade tungsten carbide bur coated with zirconium nitride (Komet, H23VIP) mounted on a blue contra-angle handpiece (low speed) was used with light pressure, under air/water spray cooling, at the optimal speed of 40,000 rpm. Then a silicone polisher (Komet, 9498) was used at the optimal speed of 6,000 rpm under air/water spray cooling. For the E-kit, two successive silicone polishers were used on a blue contra-angle under water cooling, in which the first one was with medium grains (40-50mm), and the second one with fine grains (3–6 mm), with an optimal speed comprised between 3,000 and 8,000 rpm. The enamel surface was considered clean and free of composite by visual inspection by the operator under a dental operating light. The time needed to remove each CA was recorded, and a new bur was used for each tooth. After the removal of CA, operators were asked to fill out a satisfaction questionnaire.

### 3D Profilometry

**Fig 2:**
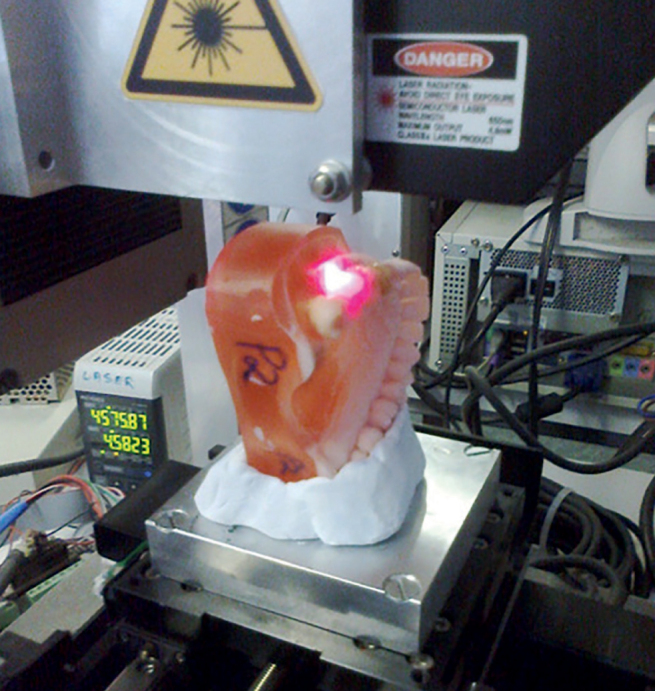
Dental arch scanning.

**Fig 3:**
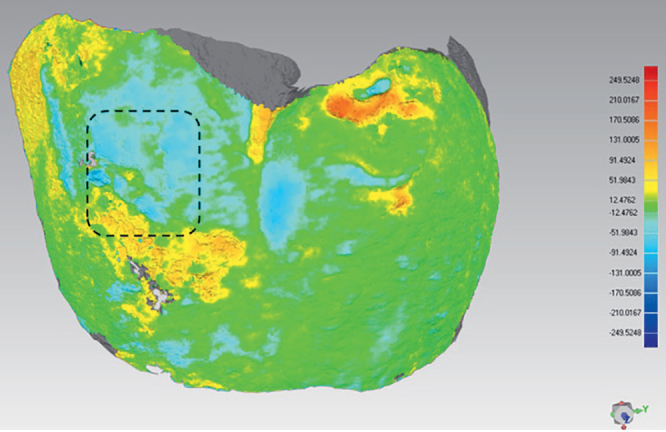
Superimposition of the T0 and T1 scans of a molar buccal surface. The dotted line delimits the area considered for the enamel surface height variation (ESHV) measurements (CAA). CAA: composite attachments area.

The dental arches were cleaned with a cotton swab and ethanol to avoid any pollution of the vestibular surfaces. They were scanned at baseline (T0) and after CA removal (T1) using a custom-made device comprising a motorized XY stage and a 100-nm resolution laser sensor (Keyence LK G30 with LK GD500 controller, Keyence Corporation, Osaka, Japan) with a step of 25-μm.^15^ The dental arches were positioned in a specific holder to ensure reproducible positioning (Fig 2). Raw data acquisition and processing were performed using custom-developed software in C# language (Microsoft Visual Studio 2013, Microsoft Corporation, Redmond, WA, USA) coupled with a digital data acquisition PCI card (NI PCI-6534, National Instruments Corporation, Austin, TX, USA). The resulting matrix of Z values at baseline (T0) and T1 were superimposed using a surface matching software (Geomagic Control 2015, Geomagic, Morrisville, NC, USA) to measure enamel surface height variation (ESHV) in areas of CA (CAA). CAA were determined by taking the templates used for creating the CA as a reference (Fig 3). Baseline scans were transformed into a computer-aided design format (STL), and T1 scans of the entire tooth were superimposed using a best-fit alignment algorithm with a threshold of minimum deviation set at 15 μm as previously described.^15^ To prevent bias in the measurement related to artifacts or surface pollution, a threshold value of 250 μm was defined, leading to the exclusion of points with a measured difference superior to 250 μm from the enamel loss evaluation. The matching was accepted when the root mean square (RMS) was less than 80 µm. Once the matching of the entire tooth is validated through this process, a measure of ESHV was obtained in CAA only (Fig 3). The accuracy threshold of the measurement chain was 15 μm.^15^ The same operator performed all of the measurements. Mean enamel loss was assessed from ESHV measurement in CAA. Enamel loss was set to zero for teeth with CAA fully covered by composite remnants and then exhibiting positive ESHV values.

### Digital Microscopy

**Fig 4:**
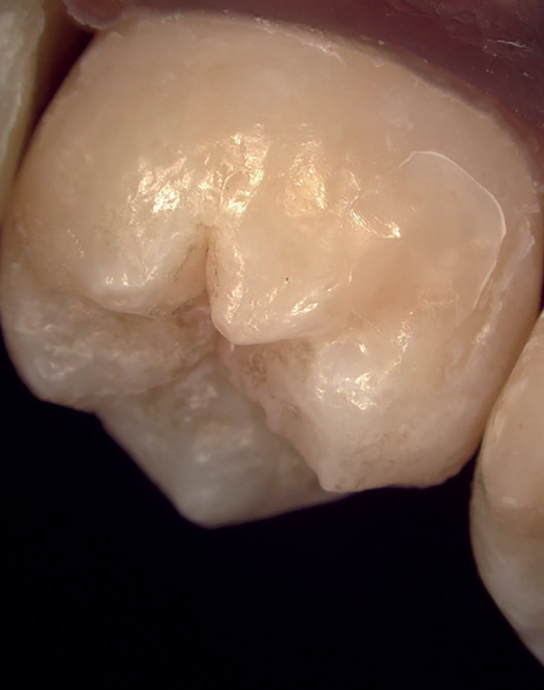
Digital microscope photograph of the tooth in position 17 of arch 2 (VHX-7000, Keyence, IL, USA).

The teeth were analyzed using digital microscopy (VHX-7000, Keyence, IL, USA) at magnification ×20 to analyze the presence of composite remnants after CA removal (Fig 4).

### Statistical Analysis

Experimental data were summarized by means, standard deviation (SD), and range. To assess the effect of experimental factors (operator, tool, dental loupes, tooth position) on study outcomes (enamel loss, presence of composite remnants, time to remove CA), data were analyzed by generalized linear mixed-effects models to account for repeatability and variability of teeth within the same dental arches. The tooth position was scored from 1 (last molar) to 4 (premolar) but also treated as a qualitative factor. Results were expressed as regression coefficients with standard error (SE) and considered significant at the 5% critical level (p < 0 .05). All calculations were performed with SAS (version 9.4).

### Pilot Study

A pilot study was conducted on two dental arches, with two operators testing the feasibility of the project and two others assessing the tools to be used.

## Results

The experiment yielded a total of 32 observations (8 arches and 4 teeth/arch) for each outcome. Distribution characteristics of study outcomes and control parameter are presented in Table 1.

**Table 1:** Distribution characteristics of study outcomes and control parameter

Variable	N	Mean ± SD	Range
Outcome			
Enamel loss (µm)	30*	–22.7 ± 29.4	–132 to 0
Presence of composite remnants (n, %)	32	11 (34.4)	
Time to remove the composite (sec)	32	70.0 ± 20.6	31.7–109.6
Control parameter			
RMS error (µm)	30*	38.9 ± 14.8	15.6–78.5

*Two teeth excluded.

### Enamel Loss

Two teeth showing significant areas of positive and negative values in the CAA, meaning an area of composite remnants and also an area of enamel damage in the CAA, were excluded from the analysis. The mean enamel loss was –22.7 ± 29.4 µm (range –132 to 0 µm). Of the 30 teeth, 10 exhibited no enamel loss, as the area of interest was entirely covered by composite remnants. Five teeth demonstrated minimal enamel loss (less than –15 µm, the precision threshold of the measuring chain). Ten teeth exhibited enamel loss between –15 and –50 µm, and five teeth exhibited enamel loss exceeding –50 µm. The application of linear mixed-effects models to the enamel data set (Table 2) revealed that none of the experimental factors was significantly associated with enamel loss.

**Table 2:** Impact of experimental factors on enamel loss (µm) assessed by linear mixed-effects modeling of data*

Factor	Coefficient ± SE	P-value
Intercept	–18.7 ± 19.1	0.40
Technique (S-Kit vs. E-Kit)	2.62 ± 14.4	0.87
Operator (junior vs. senior)	7.03 ± 20.7	0.76
Dental loupes (no vs. yes)	5.67 ± 20.0	0.80
Tooth position (1–4)	–0.48 ± 4.37	0.91
Operator × dental loupes	–38.8 ± 28.8	0.27

*Two teeth excluded (N = 30).

### Presence of Composite Remnants

Composite remnants were observed on 11 (34.4%) teeth out of 32 (Tables 1 and 3). Generalized linear mixed modeling applied to composite remnants (Table 4) evidenced a significant effect of the operator (2.7 ± 1.3, p = 0.038), with junior operators leaving less residual composite remnants than senior operators. A significant effect was also observed for the tool used (coefficient ± SE: 2.7 ± 0.76, p = 0.0005), with the Easycomp kit leaving more composite remnants than the Smoozies kit, and for dental loupes (–3.5 ± 1.3, p = 0.0090), which significantly reduced the risk of leaving a composite remnant. The presence of residual composite did not vary with tooth position (–0.32 ± 0.33, p = 0.33).

**Table 3:** Outcome characteristics according to each dental arch

Dental arch	Enamel loss (µm) Mean ± SD	Residual composite (%)	Time to remove composite (sec) Mean ± SD
1	–28.3 ± 7.4	0.0	63.2 ± 6.5
2	–8.9 ± 17.8	100.0	90.1 ± 20.3
3	–1.0 ± 1.8*	25.0	69.0 ± 10.0
4	–22.3 ± 33.3*	25.0	49.8 ± 5.2
5	–1.0 ± 2.0	75.0	98.0 ± 5.9
6	–24.9 ± 31.1	50.0	77.2 ± 11.7
7	–61.2 ± 48.1	0.0	41.7 ± 9.7
8	–28.3 ± 23.4	0.0	71.0 ± 14.2

* One tooth excluded.

**Table 4:** Impact of experimental factors on the presence of composite remnants as assessed by generalized linear mixed-effects modeling of the data (N = 32)

Factor	Regression coefficient ± SE	P-value
Intercept	–1.43 ± 1.29	0.27
Technique (S-kit vs. E-kit)	2.65 ± 0.76	0.0005
Operator (junior vs. senior)	2.65 ± 1.27	0.038
Dental loupes (no vs. yes)	–3.47 ± 1.33	0.0090
Tooth (1–4)	–0.32 ± 0.33	0.33

### Procedure Duration

The overall mean time to remove CA was 70.0 ± 20.6 sec (range: 31.7–109.6 s) as described in Table 1 and Table 3. Applying a linear mixed-effects model to the data (Table 5), two factors were statistically significant: the operator (coefficient ± SE: 19.9 ± 5.2, p = 0.031) and the interaction between operator and dental loupes (–40.3 ± 7.4, p = 0.012). Junior operators worked faster than senior operators, but the latter were quicker when using the dental loupes. Neither the tool used nor the position of the tooth affected the time to remove the composite. The latter conclusion did not change when considering the position of the teeth as a qualitative variable.

**Table 5:** Impact of experimental factors on time (sec) to remove composite attachments as assessed by linear mixed modeling of the data (N = 32)

Factor	Regression coefficient ± SE	P-value
Intercept	66.8 ± 6.1	–
Technique (S-kit vs. E-kit)	3.0 ± 3.7	0.47
Operator (junior vs senior)	19.9 ± 5.2	0.031
Dental loupes (no vs yes)	–8.0 ± 5.2	0.22
Tooth (1–4)	2.3 ± 1.8	0.21
Operator × dental loupe	–40.3 ± 7.4	0.012

The satisfaction questionnaire revealed no difference between the two kits.

## Discussion

This study was designed to simulate the clinical setting as much as possible, while allowing accurate enamel loss measurement with digital profilometry using an optical scanner. Basically, this measurement method is reported to be the most reliable,^29^ and to the best of the authors’ knowledge, it has never been used to measure enamel loss after the removal of orthodontic devices. It should be noted that anterior teeth were not used here due to the rarity of their extraction, while according to Baumann et al,^3^ molars are usually more damaged than anterior teeth during orthodontic bracket removal.

The results showed that aligner CA removal can induce enamel tissue loss and lead to the presence of composite remnants. Moderate to severe enamel loss was observed in half of the teeth, 5 teeth out of 30 showing more than 50 µm loss on average in the area. The only study that evaluated enamel loss after orthodontic bracket removal using contact profilometry reported values around –50 µm with either tungsten carbide or diamond disks.^27^ Those values are higher than the mean enamel loss observed in this study, which can be explained by the method used and the type of orthodontic device (brackets versus aligner CA). Here, the operator’s experience (junior versus senior), the tool, and the tooth position did not influence enamel loss. It is important to note, however, that the presence of composite remnants results in a reduction of the enamel loss sample, which in turn limits the capacity to investigate the subtle influence of the experimental factors.

Indeed, composite remnants were found in 34.4% of teeth and significantly more in senior than in junior operators (p = 0.038). They were more frequent with silicone polishers than with tungsten carbide burs (p = 0.0005) and were reduced using dental loupes (p = 0.0090). Baumann et al^3^ also showed that the presence of composite remnants after bracket debonding is reduced by using dental loupes. Indeed, Bernard^4^ demonstrated that the size of the smallest detail visible to the human eye was 50 µm. Composite remnants should be completely removed from the tooth surface so they can be restored to their original state as closely as possible. As explained by Tekçe et al,^26^ composites are submitted to shade alterations over time, which can have negative esthetic outcomes. In addition, composite remnants are elevated zones that can lead to the formation of biofilm and promote caries.^6,9,13,16^ Ryf et al^24^ found resin remnants in 27% of teeth, close to the 34.4% reported in the present study. According to these authors, if the surface appears lustrous, it tends to prevent the clinician from further polishing. Indeed, the operators left more composite when using the Easycomp kit, which tends to make the surface very shiny. Rocha et al^21^ observed fewer residues when using fluorescent lighting to visualize the composite during removal. Ideally, it is preferable to remove all the composite while limiting enamel loss. In the present study, among teeth without composite remnants, 25% had little enamel loss (<15 µm, the accuracy threshold of the measuring chain) and may be considered null.

Finally, time is an important factor in orthodontics, and, contrary to Mohebi et al,^17^ the present experimental study indicated that dental loupes also reduce working time when removing CA. Soares Tenório et al^25^ found a significant time reduction using a tungsten carbide bur compared to a silicon polisher. In the present study, this time reduction was not significant Furthermore, tungsten carbide burs are more aggressive toward enamel and should never come into direct contact with it. The use of dental loupes is also recommended with this type of bur to leave a thin layer of composite, which can then be removed with a silicon polisher. However, the involvement of different operators using or not using magnification loupes may have introduced a potential but unavoidable bias in the study. Indeed, operators accustomed to a specific system may find it challenging to work without magnification loupes if they are accustomed to using them, and vice versa. The reliance on a single operator accustomed to a particular working style, with or without loupes, may limit the generalizability of the present experimental study findings. This inherent bias underscores the complexities associated with comparing outcomes between operators who have specific habits and preferences regarding the use of magnification tools in their dental procedures. Despite this limitation, the study has drawn meaningful insights within the constraints of this inherent variability, recognizing the potential impact on the results due to operator-specific factors.

## Clinical relevance:

This experimental study showed that CA removal can lead to enamel loss and to the presence of composite remnants. Although enamel loss was not influenced by the operator’s experience, the type of tool or tooth position, the presence of composite remnants could be reduced by using proper tools and dental loupes. Additionally, dental loupes helped to decrease the working time.

## References

[ref1] Ahrari F, Akbari M, Akbari J, Dabiri G (2013). Enamel surface roughness after debonding of orthodontic brackets and various clean-up techniques. J Dent (Tehran).

[ref2] Alessandri Bonetti G, Zanarini M, Incerti Parenti S, Lattuca M, Marchionni S, Gatto MR (2011). Evaluation of enamel surfaces after bracket debonding: an in-vivo study with scanning electron microscopy. Am J Orthod Dentofacial Orthop.

[ref3] Baumann DF, Brauchli L, Waes H van (2011). The influence of dental loupes on the quality of adhesive removal in orthodontic debonding. J Orofac Orthop.

[ref4] Bernard H (2020). Regard sur l’image: L’œil humain, précisions et échelles de grandeur V6. https://www.regard-sur-limage.com/precisions-et-echelle-de-grandeur-oeil-humain.html.

[ref5] Cehreli ZC, Lakshmipathy M, Yazici R (2008). Effect of different splint removal techniques on the surface roughness of human enamel: a three‐dimensional optical profilometry analysis. Dent Traumatol.

[ref6] Chen Q, Zheng X, Chen W, Ni Z, Zhou Y (2015). Influence of orthodontic treatment with fixed appliances on enamel color: a systematic review. BMC Oral Health.

[ref7] Eliades T (2004). Enamel surface roughness following debonding using two resin grinding methods. Eur J Orthod.

[ref8] Erdur EA, Akin M, Cime L, Ileri Z (2016). Evaluation of enamel surface roughness after various finishing techniques for debonding of orthodontic brackets. Turk J Orthod.

[ref9] Ferracane JL (2016). Models of caries formation around dental composite restorations. J Dent Res.

[ref10] Garg R, Dixit P, Khosla T, Gupta P, Kalra H, Kumar P (2018). Enamel surface roughness after debonding: a comparative study using three different burs. J Contemp Dent Pract.

[ref11] Grocholewicz K (2014). Effect of Orthodontic debonding and adhesive removal on the enamel – current knowledge and future perspectives – a systematic review. Med Sci Monit.

[ref12] Howell S, Weekes WT (1990). An electron microscopic evaluation of the enamel surface subsequent to various debonding procedures. Aust Dent J.

[ref13] Joo H-J, Lee Y-K, Lee D-Y, Kim Y-J, Lim Y-K (2011). Influence of orthodontic adhesives and clean-up procedures on the stain susceptibility of enamel after debonding. Angle Orthod.

[ref14] Karan S, Kircelli BH, Tasdelen B (2010). Enamel surface roughness after debonding: comparison of two different burs. Angle Orthod.

[ref15] Koenig V, Wulfman C, Bekaert S, Dupont N, Le Goff S, Eldafrawy M, Vanheusden A, Mainjot A (2019). Clinical behavior of second-generation zirconia monolithic posterior restorations: two-year results of a prospective study with ex vivo analyses including patients with clinical signs of bruxism. J Dent.

[ref16] Moecke SE, Barros PCA, Andrade ACM, Borges AB, Pucci CR, Torres CRG (2022). Efficacy of bracket adhesive remnant removal by a fluorescence-aided identification technique with a UV light handpiece: in vitro study. Int J Dent.

[ref17] Mohebi S, Shafiee H-A, Ameli N (2017). Evaluation of enamel surface roughness after orthodontic bracket debonding with atomic force microscopy. Am J Orthod Dentofacial Orthop.

[ref18] Oliver RG, Griffiths J (1992). Different techniques of residual composite removal following debonding – time taken and surface enamel appearance. Br J Orthod.

[ref19] Putrino A, Barbato E, Galluccio G (2021). Clear aligners: between evolution and efficiency – a scoping review. Int J Environ Res Public Health.

[ref20] Retief DH, Denys FR (1979). Finishing of enamel surfaces after debonding of orthodontic attachments. Angle Orthod.

[ref21] Rocha RS, Salomão FM, Silveira Machado L, Sundfeld RH, Fagundes TC (2016). Efficacy of auxiliary devices for removal of fluorescent residue after bracket debonding. Angle Orthod.

[ref22] Rosin M, Splieth C, Hessler M, Gärtner C, Kordaß B, Kocher T (2002). Quantification of gingival edema using a new 3‐D laser scanning method. J Clin Periodontol.

[ref23] Rouleau BD, Marshall GW, Cooley RO (1982). Enamel surface evaluations after clinical treatment and removal of orthodontic brackets. Am J Orthod.

[ref24] Ryf S, Flury S, Palaniappan S, Lussi A, Meerbeek B van, Zimmerli B (2011). Enamel loss and adhesive remnants following bracket removal and various clean-up procedures in vitro. Eur J Orthod.

[ref25] Soares Tenório KC, Neupmann Feres MF, Tanaka CJ, Augusto MKM, Rodrigues JA, Silva HD Pereira da, Arana-Chavez VE, Roscoe MG (2020). In vitro evaluation of enamel surface roughness and morphology after orthodontic debonding: traditional cleanup systems versus polymer bur. Int Orthod.

[ref26] Tekçe N, Tuncer S, Demirci M, Serim ME, Baydemir C (2015). The effect of different drinks on the color stability of different restorative materials after one month. Restor Dent Endod.

[ref27] Tüfekçi E, Merrill TE, Pintado MR, Beyer JP, Brantley WA (2004). Enamel loss associated with orthodontic adhesive removal on teeth with white spot lesions: an in vitro study. Am J Orthod Dentofacial Orthop.

[ref28] Vidor MM, Felix RP, Marchioro EM, Hahn L (2015). Enamel surface evaluation after bracket debonding and different resin removal methods. Dent Press J Orthod.

[ref29] Wulfman C, Koenig V, Mainjot AK (2018). Wear measurement of dental tissues and materials in clinical studies: a systematic review. Dent Mater.

[ref30] Zachrisson BU, Årthun J (1979). Enamel surface appearance after various debonding techniques. Am J Orthod.

[ref31] Zarrinnia K, Eid NM, Kehoe MJ (1995). The effect of different debonding techniques on the enamel surface: an in vitro qualitative study. Am J Orthod Dentofacial Orthop.

